# Supplementary material for: Natural and engineered mediators of desiccation tolerance stabilize Human Blood Clotting Factor VIII in a dry state
Source: Sci Rep. 2023 Mar 20;13:4542. doi: 10.1038/s41598-023-31586-9 (PMC10027729; doi:10.1038/s41598-023-31586-9)
Supplement: Supplementary file 1 — Supplementary Information. [file 41598_2023_31586_MOESM1_ESM.docx]

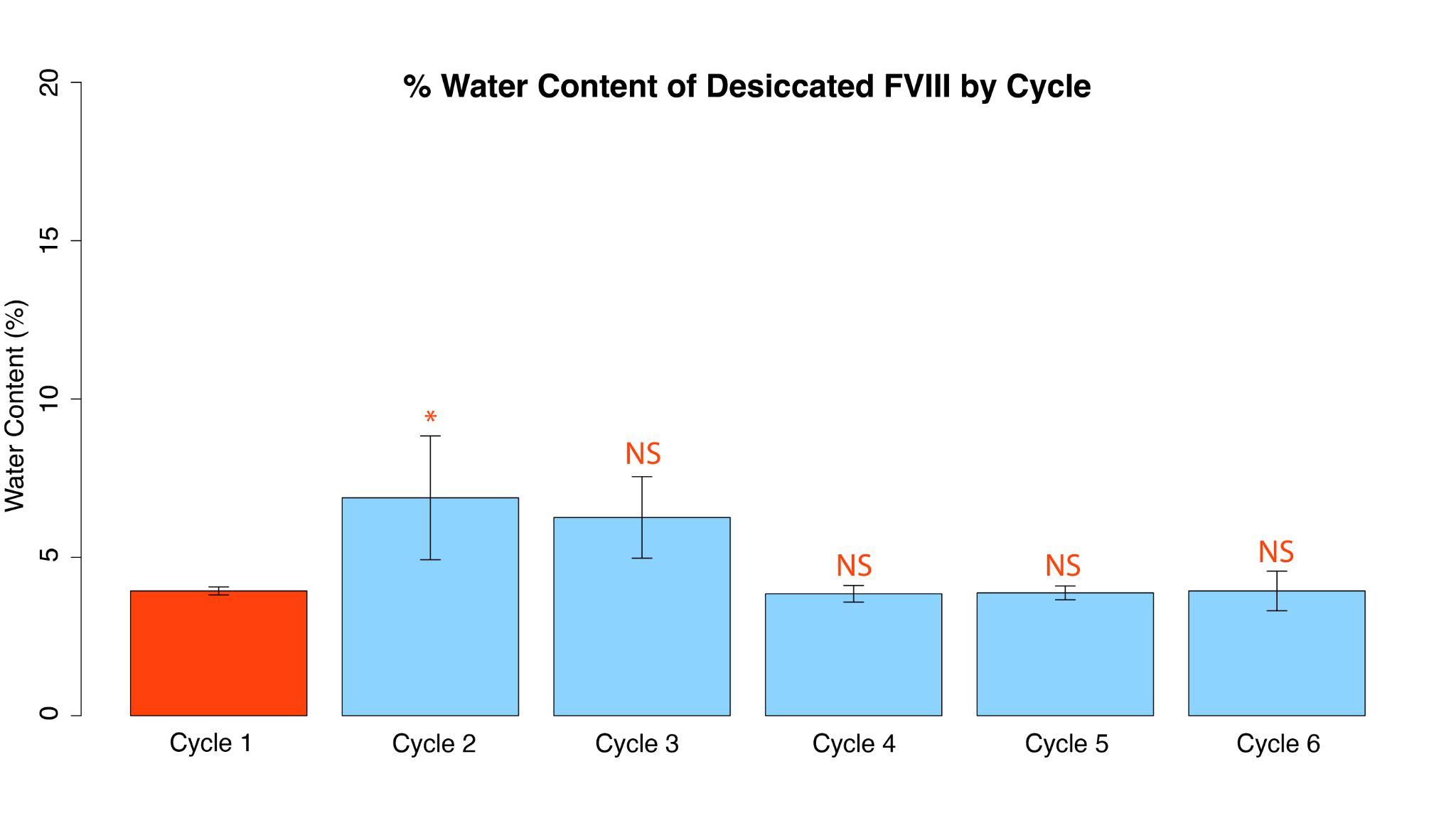


Figure S1 **Water content of dried FVIII during repeated desiccation cycles.** Water content of dried FVIII samples after 1, 2, 3, 4, 5, or 6 desiccation/rehydration cycles (n = 3). Error bars represent bi-directional standard deviation. Notations above sample bars represent statistical significance determined by one-way ANOVA and Tukey’s post- hoc test. P value > 0.05 = NS, P < 0.05 = *, P < 0.01 = **, P < 0.001 = ***
